# Is a multispecies probiotic mixture effective in constipation during pregnancy? 'A pilot study'

**DOI:** 10.1186/1475-2891-11-80

**Published:** 2012-10-04

**Authors:** Inge de Milliano, Merit M Tabbers, Joris A van der Post, Marc A Benninga

**Affiliations:** 1 Department of Paediatric Gastroenterology and Nutrition, Emma Children’s Hospital/ Academic Medical Centre, University of Amsterdam, Amsterdam, The Netherlands; 2 Department of Obstetrics and Gynaecology, Academic Medical Centre, University of Amsterdam, Amsterdam, The Netherlands

**Keywords:** Probiotics, Constipation, Pregnancy

## Abstract

**Background:**

Constipation during pregnancy is a common problem. Nowadays only few effective interventions are published preventing or treating constipation during pregnancy. However, their use is limited due to side-effects. This uncontrolled intervention study was performed to determine if a mixture of probiotics in the treatment of constipation during pregnancy is effective.

**Methods:**

Women aged ≥ 18 years with functional constipation were included at the Obstetrical outpatient clinic and midwife practices. Patients received during four weeks a daily dose of Ecologic®Relief (*Bifidobacterium bifidum* W23*, Bifidobacterium lactis* W52*, Bifidobacterium longum* W108*, Lactobacillus casei* W79*, Lactobacillus plantarum* W62 and *Lactobacillus rhamnosus* W71 (total 4*10^9^ CFU)). For all analyses, the non-parametric paired Wilcoxon test was used. Primary outcome measure was change in defecation frequency. Secondary outcome measures were stool consistency, sensation of incomplete evacuation, sensation of anorectal obstruction, manual manoeuvres to facilitate defecation, abdominal pain, adverse effects, presence of reflux episodes and intake of Bisacodyl.

**Results:**

20 women were included. Defecation frequency significantly increased from 3.1 at baseline to 6.7 in week four (p < 0.01). Compared to baseline, a significant decrease in 1) sensation of anorectal obstruction from 90.0% to 45.0% (p < 0.01), 2) sensation of incomplete evacuation from 90.0% to 40.0% (p < 0.01), 3) straining during defecation from 100% to 65% (p = 0.01), 4) episodes of abdominal pain from 60% to 20% (p = 0.01) and 5) the presence of reflux episodes from 60% to 20% in week four (p = 0.01) was found. Other secondary outcomes did not decrease significantly. No side effects were reported.

**Conclusions:**

Ecologic®Relief is effective in the treatment of constipation during pregnancy. A randomised placebo controlled trial is required to confirm these data.

## Background

The reported prevalence of constipation in pregnant women varies between 11-38% and occurs mostly during the third trimester, although symptoms can also be present from 12 weeks gestation [1-4]. According to the ROME III criteria constipation is characterised by straining during defecation, lumpy or hard stools, sensation of incomplete evacuation or anorectal obstruction, manual manoeuvres to facilitate defecation, and/or less than three defecations per week [5]. At physical examination, a palpable faecal mass is often found in the abdomen or the rectum [6,7].

The pathophysiology underlying functional constipation is undoubtedly multifactorial and not well understood. Progressively rising progesterone and estrogen levels have been suggested as cause of constipation during pregnancy [3,8,9]. Animal studies have shown that these female hormones, particularly progesterone, inhibit gut smooth muscle thereby decreasing esophageal, gastric, and colonic muscle contractility [3,10]. Low fluid and fibre intake may also play a role. It has been suggested that pregnant women consume less fibres than recommended for the non-pregnant population, however this assumption has not yet been confirmed [11,12].

Nowadays, there are few effective interventions published preventing or treating constipation during pregnancy. There is weak evidence showing that increased fibre intake treating constipation during pregnancy may improve constipation by increasing the defecation frequency as compared to placebo [13]. A recent published review evaluating the effectiveness of different laxatives, concluded that stimulant laxatives may be more effective than bulk laxatives in improving constipation during pregnancy by increasing bowel movements and softening the stools [14]. However, their adverse effects, such as abdominal pain and diarrhoea, limit their use. In addition, these studies incorporate low-quality evidence [14].

Therefore, alternatives that are safe for pregnant women and the fetus are needed. These alternatives must be in concordance with the guidelines for treatment of constipation during pregnancy, stating that non-pharmacological measures are the first step in treatment [15]. Probiotics are defined as live micro-organisms which when administered in adequate amounts confer a health benefit on the host [16]. Probiotics probably have at least two modes of action in improving constipation. Firstly, it is assumed that dysbiosis in the gut flora plays a role in constipation. Probiotics might improve this dysbiosis [17,18]. Secondly, probiotics are able to lower pH of the colon by producing lactic, acetic and other short chain fatty acids. A lower pH enhances colonic peristalsis and subsequently decreases colonic transit time [17,18]. Probiotics might also be effective in the treatment of constipated pregnant women. Probiotics have shown to be safe for mother and fetus during pregnancy. Several randomised controlled trials to investigate the safety of probiotics during pregnancy did not report an increase in adverse events related to probiotics [17-23]. Furthermore, current data suggest that probiotic supplementation is rarely systemically absorbed when used by healthy individuals [23].

Few studies on the effectiveness of probiotics in constipated adults have been performed. A recent review found that strains of lactobacilli and bifidobacteria increased the defecation frequency and improved stool consistency in constipated men and women [24]. In addition, a randomised controlled trial in constipated elderly using *Lactobacillus rhamnosus* and *Propionibacterium freudenreichii* showed positive results in defecation frequency [25]. Other randomised controlled trials in constipated adults also show significant effects on defecation frequency and stool consistency [26-28]. Bekkali at al. [29] described the effectiveness of Ecologic®Relief (a mixture of *Bifidobacterium bifidum* W23*, Bifidobacterium lactis* W52*, Bifidobacterium longum* W108*, Lactobacillus casei* W79*, Lactobacillus plantarum* W62 and *Lactobacillus rhamnosus* W71) in constipated children. Based on the positive results of probiotics in constipated children and the adverse effects of existing treatment options for constipation during pregnancy, we hypothesized similar positive effects of this probiotic mixture in constipated pregnant women not responding to conventional treatments. Therefore we performed a pilot study to determine whether this mixture is effective on constipation during pregnancy.

## Methods

### Subjects

Pregnant women with constipation aged ≥ 18 years, who are between 12 and 34 weeks into their pregnancy were eligible. Our goal was to include 20 pregnant women. They were included at the Obstetrical outpatient clinic of the Academic Medical Centre in Amsterdam and 28 participating midwife practices in the municipal of Amsterdam. Women were included when they were suffering from functional constipation according to the Rome III criteria [5] (Table 1). Criteria had to be fulfilled for at least two weeks with symptom onset during pregnancy. Women were excluded if they had been treated for constipation less than one week before the start of the study. Other exclusion criteria were: a diagnosis of mental retardation or metabolic disease (hypothyroidism), Hirschsprung’s disease, spinal anomalies, anorectal pathology, inflammatory bowel disease, previous gastrointestinal surgery, the use of fermented dairy products containing probiotics two weeks prior to the study. All participants signed an informed consent. This pilot was approved by the medical ethical committee of the Academic Medical Centre, Amsterdam.

**Table 1:** Rome III criteria for functional constipation


I. At least 2 or more of the following criteria:
-Straining during at least 25% of the defecations
-Lumpy or hard stools in at least 25% of the defecations
-Sensation of incomplete evacuation for at least 25% of defecations
-Sensation of anorectal obstruction/blockage for at least 25% of defecations
-Manual manoeuvres to facilitate defecation for at least 25% of defecations
-Fewer than three defecations per week
II. Loose stools are rarely present without the use of laxatives
III. Insufficient criteria for irritable bowel syndrome

### Study design

This pilot study was an uncontrolled intervention study intended to evaluate feasibility, adverse effects and effect size of the probiotics in constipated pregnant women. All subjects received four grams of Ecologic®Relief containing (4*10^9^ CFU) of the probiotic strains *Bifidobacterium bifidum* W23*, Bifidobacterium lactis* W52*, Bifidobacterium longum* W108*, Lactobacillus casei* W79*, Lactobacillus plantarum* W62 and *Lactobacillus rhamnosus* W71 daily for four weeks. Bekkali et al. [29] showed four weeks is an appropriate period to observe short-term (adverse) effects of probiotics. Other components of this powder were very small amounts of rice starch, maltodextrins, inulin, potassium chloride, magnesium sulphate, fructo-oligosaccharides (FOS) and manganese sulphate. Patients were instructed to mix the powder in one glass (200 mL) of lukewarm water in the morning and ingest this solution before breakfast.

At intake, all participants completed a non-validated questionnaire created by the authors about their defecation frequency, consistency of stools, sensation of incomplete evacuation or anorectal obstruction and manual manoeuvres regarding the week prior to the intake. Also gastro-intestinal symptoms such as abdominal pain and reflux episodes were evaluated. Furthermore, a general medical history and the use of medication were listed. Information and education about functional constipation was given to all patients. Before the start of the probiotic treatment, all subjects received one rectal enema (Microlax) once daily for three days in order to achieve rectal disimpaction to create a homogeneous study group.

If a subject did not defecate for three days during the study period, a stimulant laxative (Bisacodyl 5 mg, orally or rectally) was prescribed on the fourth day. During the study, women were not allowed to consume any fermented dairy products containing probiotics or any laxatives, except for the rescue medication Bisacodyl. Participants were asked not to change their daily dietary intake, like fluids and fibres, during the study period.

During the treatment phase intake of study medication, defecation frequency, consistency of stools, pain during defecation, frequency of episodes of faecal incontinence, sensation of incomplete evacuation or anorectal obstruction and possible adverse effects such as abdominal pain, diarrhoea and bad taste were recorded daily in a defecation diary by the pregnant women. This diary contained similar questions as the questionnaire used at baseline. After four weeks, the study medication was stopped and a follow-up appointment was scheduled two weeks later.

Clinical evaluation, frequency of adverse effects and compliance were evaluated at baseline and at two, four and six weeks after starting probiotic usage.

### Outcome measures

The primary outcome measure was the change in defecation frequency after four weeks of probiotic use compared to baseline. Secondary outcome measures were stool consistency, sensation of incomplete evacuation, sensation of anorectal obstruction, manual manoeuvres to facilitate defecation, abdominal pain, adverse effects (bad taste and diarrhoea), presence of reflux episodes and frequency of intake of Bisacodyl.

### Analysis

Baseline characteristics, adverse effects and Bisacodyl use were documented in a descriptive way. For change in defecation frequency and for the comparison of all secondary outcome measures between baseline and the evaluation time points, the non-parametric paired Wilcoxon test was used. Data at baseline were weekly outcomes, therefore data from the defecation diary were summarized into weekly outcomes in order to be able to compare. Statistical significance for the primary outcome measure and all secondary outcome measures was defined as p ≤ 0.05. All analyses were performed in SPSS (version 17.0).

## Results

Between October 2008 and May 2011, 20 women were enrolled into this pilot study and all participants completed the study. The baseline characteristics are summarized in Table 2.

**Table 2:** Baseline patient characteristics: median (SD), n = 20

	
Age in years	29.5 (5.3)
Gravidity	1.0 (1.3)
Parity	0.0 (0.4)
Number of weeks pregnant	19.5 (7.5)
Stool frequency per week	3.0 (1.6)
Straining during defecation	100.0%
Hard stools	90.0%
Sensation of incomplete evacuation	90.0%
Sensation of anorectal obstruction	90.0%
Manual manoeuvres	10.0%
Abdominal pain	60.0%
Reflux episodes	60.0%

**Figure 1:**
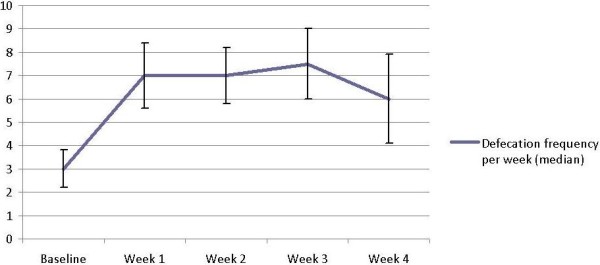
Primary outcome: change in defecation frequency (n=20), p < 0.01.

The median defecation frequency per week significantly increased from 3.0 at baseline to 7.0 in week two (p < 0.01) and 6.0 in week four (p < 0.01) (Figure1). As shown in Table 3, all secondary outcome measures, except for hard stools and manual manoeuvres, improved significantly after two weeks and these results sustained until the end of the treatment period.

**Table 3:** Secondary outcome measures with p-values (n = 20)

**Outcome**	**Baseline**	**Week 2**	**Week 4**	**P-value (week 4 – week 0)**
Straining during defecation	100.0%	70.0%	65.0%	P = 0.01
Hard stools	90.0%	65.0%	65.0%	P = 0.10
Sensation of incomplete evacuation	90.0%	35.0%	40.0%	P < 0.01
Sensation of anorectal obstruction	90.0%	40.0%	45.0%	P < 0.01
Manual manoeuvres	10.0%	5.0%	5.0%	P = 0.56
Abdominal pain	60.0%	30.0%	20.0%	P = 0.01
Reflux episodes	60.0%	45.0%	20.0%	P = 0.01

Rescue medication, Bisacodyl, was used by two women. One patient used Bisacodyl once in week one, five times in week two and daily in week three and week four. The second woman used Bisacodyl only once in week four. No side effects were reported. Based on the diary, the compliance was 100%.

## Discussion

To our knowledge, this is the first study investigating the use of probiotics in constipated pregnant women. A significant increase in defecation frequency was observed. Furthermore, the presence of sensation of incomplete evacuation, sensation of anorectal obstruction, straining during defecation, abdominal pain and reflux episodes also improved significantly with the use of this probiotic mixture. No adverse effects were reported during the study.

The improvement of all important defecation characteristics in constipated pregnant women are in accordance with the results found by Bekkali et al. in 20 children with constipation using the same mixture of probiotics. The latter study reported a significant increase in bowel movements, a decrease in fecal incontinence episodes and a decrease in abdominal pain [29].

Our results are also in line with a recent review on the efficacy of probiotics by Chmielewska et al. [24]. They found that *Bifidobacterium lactis* DN-173 010, *Lactobacillus casei* Shirota and *Escheria Coli* Nissle 1917, used in different randomised controlled trials in constipated men and non-pregnant women, increased the defecation frequency and improved stool consistency [24]. A recent double-blind randomised placebo controlled trial in otherwise healthy constipated adults using different strains of lactobacilli and bifidobacteria also showed an increase in bowel movements, positive effects on stool consistency and on discomfort items such as abdominal bloating and anal itching [26]. A randomised trial in constipated elderly using a mixture of probiotics including *Lactobacillus rhamnosus* and *Propionibacterium freudenreichii* showed positive results with respect to defecation frequency as well [25]. It should be noted that the composition of the strains of probiotics used in the described studies is different and effects might therefore be strain- or product-specific. For this reason, one should be careful comparing these studies. Studies comparing the effects of these different strains are not available.

It has been suggested that the findings in men and non-pregnant women, elderly and children are not directly applicable to constipated pregnant women, because of differences in pathophysiology. The main difference is the changed hormone levels during pregnancy (*e.g.* progesterone) [3,8,9,11,12]. Our study was designed as a pilot study on the use of probiotics and not to elucidate the effect of probiotics on the different pathophysiological mechanisms in constipation. So, this hypothesis remains unresolved. The fact that our results are in line with published results in non-pregnant women, suggests that at least some of the pathophysiological mechanisms are independent of pregnancy and comparable to non-pregnancy, like low fibre and low fluid intake.

### Clinical relevance

Pregnant women may experience constipation for the first time during pregnancy or their existing constipation symptoms increase in severity during pregnancy [30]. Besides discomfort of constipation symptoms, straining during defecating can damage the pudendal nerve and impair the supportive function of the pelvic floor musculature [31,32]. Furthermore disturbed defecation can result in the development of uterovaginal prolapse [33]. Therefore it is important to recognize the symptoms of constipation and treat these complaints in an early phase of pregnancy. In clinical practice, laxatives and fibres are frequently used. Laxatives like Lactulose and Bisacodyl have proven to be safe for mother and fetus and should therefore be considered in the treatment of constipation during pregnancy [14]. Unfortunately, their side effects, such as abdominal pain and diarrhoea, limit their use [14]. Besides, changing dietary habits is difficult to accomplish and laxatives are considered pharmacological treatment and are not recommended as the first step in treatment of constipated pregnant women [15]. Polyethylene glycol (PEG)-based laxatives technically meet the criteria for the ideal treatment in constipation [34]. However, present data are insufficient to exclude any adverse effects on the foetus [34]. Probiotics are non-pharmacological and widely used in an otherwise healthy population. Based on the results of our study, probiotics could be of additional value in the treatment of constipation in clinical practice in pregnant women.

This study was designed as a pilot study with inherent limitations. Due to the lack of a control group, an important placebo effect of probiotics cannot be excluded. Besides that, because of the uncontrolled design, there is no information available on the natural course of constipation during pregnancy. However, since this study shows positive results on constipation symptoms, it is worthwhile to perform a large randomised trial to unravel the efficacy of this mixture probiotic strains in constipated pregnant women.

It was difficult to include the 20 patients needed for this pilot study. We experienced that underreporting of defecation problems is a major issue due to a taboo among pregnant women, midwives and doctors to discuss defecation patterns. In addition, most pregnant women who refused participation to our study, did not experience constipation as a medical problem during their pregnancy, but believed constipation is part of being pregnant. Another frequently heard reason for not participating in the study is that pregnant women experience their pregnancy as a stressful time and do not want to worsen this experience by participating in scientific research.

Another limitation of this study is the non-validated questionnaire used at baseline, which could have caused recall bias resulting in possible underreporting of defecation frequency per week and other defecation related parameters prior to the study. This could partly explain the initial increase in defecation frequency as shown in Figure1. But we assume the main effect observed in this study could be contributed to the mixture of probiotics. Besides, Pamuk et al. showed that the recall bias in questionnaires used to report defecation pattern is negligibly low [35].

The powder used in this study contains very small amounts of inuline and fructooligosaccharides (FOS). These inuline-type prebiotics are considered bifidogenic, stimulating the growth of *Bifidobacterium* species in the gut [36]. Studies on the effect of these prebiotics on defecation frequency and consistency of stools, show conflicting results [37]. However, it cannot be excluded that small amounts of prebiotics used in this study do have an additive effect on our results in the improvement of constipation symptoms. Future studies should address this issue.

## Conclusion

This small pilot study showed that the multispecies probiotic mixture Ecologic®Relief seems to be safe and possibly effective in constipation during pregnancy. A large placebo-controlled randomised trial is now required to confirm these data.

## Abbreviations

CFU: Colony forming units; FOS: Fructooligosaccharides; PEG: Polyethylene glycol.

## Competing interests

The authors declare that they have no competing interests.

## Authors' contributions

IDM, MMT and MAB participated in the design of the study. IDM, MMT collected the data. IDM did the statistical analysis. IDM and MMT drafted the first manuscript. All authors read and approved the final manuscript.
